# Identification of TAX2 peptide as a new unpredicted anti-cancer agent

**DOI:** 10.18632/oncotarget.4025

**Published:** 2015-05-22

**Authors:** Albin Jeanne, Emilie Sick, Jérôme Devy, Nicolas Floquet, Nicolas Belloy, Louis Theret, Camille Boulagnon-Rombi, Marie-Danièle Diebold, Manuel Dauchez, Laurent Martiny, Christophe Schneider, Stéphane Dedieu

**Affiliations:** ^1^ Université de Reims Champagne-Ardenne, Laboratoire SiRMa, UFR Sciences Exactes et Naturelles, Reims, France; ^2^ CNRS UMR 7369, Matrice Extracellulaire et Dynamique Cellulaire, MEDyC, Reims, France; ^3^ SATT Nord, Lille, France; ^4^ Université de Strasbourg, CNRS UMR 7213, Illkirch, France; ^5^ Institut des Biomolécules Max Mousseron (IBMM), CNRS UMR 5247, Université de Montpellier, Ecole Normale Supérieure de Chimie de Montpellier, Faculté de Pharmacie, Montpellier, France; ^6^ Plateforme de Modélisation Moléculaire Multi-échelle (P3M), Université de Reims Champagne-Ardenne, Reims, France; ^7^ CHU de Reims, Laboratoire Central d'Anatomie et de Cytologie Pathologiques, Reims, France

**Keywords:** TSP-1, CD47, CD36, cancer, angiogenesis

## Abstract

The multi-modular glycoprotein thrombospondin-1 (TSP-1) is considered as a key actor within the tumor microenvironment. Besides, TSP-1 binding to CD47 is widely reported to regulate cardiovascular function as it promotes vasoconstriction and angiogenesis limitation. Therefore, many studies focused on targeting TSP-1:CD47 interaction, aiming for up-regulation of physiological angiogenesis to enhance post-ischemia recovery or to facilitate engraftment. Thus, we sought to identify an innovative selective antagonist for TSP-1:CD47 interaction. Protein-protein docking and molecular dynamics simulations were conducted to design a novel CD47-derived peptide, called TAX2. TAX2 binds TSP-1 to prevent TSP-1:CD47 interaction, as revealed by ELISA and co-immunoprecipitation experiments. Unexpectedly, TAX2 inhibits *in vitro* and *ex vivo* angiogenesis features in a TSP-1-dependent manner. Consistently, our data highlighted that TAX2 promotes TSP-1 binding to CD36-containing complexes, leading to disruption of VEGFR2 activation and downstream NO signaling. Such unpredicted results prompted us to investigate TAX2 potential in tumor pathology. A multimodal imaging approach was conducted combining histopathological staining, MVD, MRI analysis and μCT monitoring for tumor angiography longitudinal follow-up and 3D quantification. TAX2 *in vivo* administrations highly disturb syngeneic melanoma tumor vascularization inducing extensive tumor necrosis and strongly inhibit growth rate and vascularization of human pancreatic carcinoma xenografts in nude mice.

## INTRODUCTION

Besides its architectural role, extracellular matrix (ECM) is commonly known to be involved in a wide range of physiological and pathological functions [1], with cell-ECM interaction actively taking part in cancer progression [2]. ECM is composed of a hydrated ground substance of glycosaminoglycans and proteoglycans, in which fibrous proteins and associated glycoproteins are embedded. Among them, thrombospondins (TSPs) consist of a family of five members with TSP-1 first identified in 1978 from human blood platelets [3]. TSP-1 is a ubiquitously expressed multimodular protein of high molecular weight secreted as a disulfide-linked 450 kDa homotrimer. The ability of TSP-1 to bind a wide variety of ligands such as cell membrane receptors or ECM molecules allows it to mediate cell-cell and cell-ECM interactions [4], thus conferring multifaceted functionalities. TSP-1 modulates tumor cell adhesion [5], proliferation [6], survival or apoptosis [7–9]. Furthermore, TSP-1 is also involved in inflammation, immune response [10] and is widely known as an endogenous inhibitor of angiogenesis [11–14] by interacting with stromal cells.

The globular carboxy-terminal cell-binding domain (CBD) of TSP-1 binds the cell surface receptor CD47, also called IAP (integrin-associated protein) [15]. CD47 is a ubiquitous 50 kDa five-spanning membrane receptor that belongs to the immunoglobulin superfamily. While TSP-1:CD47 interaction is identified as a key signaling integrator of tumor progression [4], its contribution remains somewhat controversial as TSP-1 exhibits a pleiotropic activity within the tumor microenvironment. TSP-1 is overexpressed in tumor stroma [16] and could sustain cancer cell invasion by increasing ECM-associated proteolytic activity through CD47 ligation [17]. Moreover, TSP-1 was reported to inhibit apoptosis and to promote drug resistance in thyroid carcinoma cells [7, 8] and was associated with poor prognosis and recurrence in several cancers [18–20]. However, concerns should be raised about a possible pro-tumor role for TSP-1, as a number of contrasting results were obtained considering other cancer types. Indeed, TSP-1 is down-regulated in taxane resistant cells due to its ability to induce apoptosis *via* CD47 ligation [21], and TSP-1 binding to CD47 is also recognized to induce killing of breast cancer cells [9]. Besides, TSP-1 could delay tumor growth by indirectly altering tumor blood flow [22]. Finally, TSP-1 as well as proteolytic fragments or synthetic peptides derived from TSP-1 have anti-angiogenic and anti-neoplasic potentiality [16, 23, 24].

In addition to its major contribution in cancer progression [7, 8, 16, 17, 21], TSP-1 appears to be highly implicated in the regulation of cardiovascular functions as it promotes vasoconstriction and limits angiogenesis [11, 12]. The anti-angiogenic activity of TSP-1 is mainly mediated *via* binding to CD47, resulting in disruption of CD47 association with VEGFR2 [14] and inhibition of NO-induced activation of guanylate cyclase [25]. Therefore, particular attention was paid over recent years to identify new therapeutic tools antagonizing TSP-1:CD47 in the purpose of restoring blood flow and tissue perfusion. Indeed, antibody blockade of CD47 as well as morpholino suppression of CD47 expression yields promising results regarding graft reperfusion and survival, as demonstrated by pre-clinical trials performed in mouse [26], rat [27] and porcine [28] models.

We previously characterized the molecular interface between TSP-1 CBD and CD47 using a molecular docking approach [29] since the crystallographic structure of this complex has not been resolved so far. In the present study, original molecular modeling approach led to the design of a new peptide mimicking this region and aiming to functionally antagonize the protein:protein interaction. Contrary to our initial expectation, this peptide exhibited anti-angiogenic properties *in vitro* and *ex vivo* by inhibiting TSP-1 binding to CD47 in primary endothelial cells. This unexpected result prompted us to investigate the biological consequences of peptide treatment in the context of tumor pathology. Using multimodal and multi-scale imaging approaches from *in silico* to *in vivo*, we evidenced that this CD47-derived peptide disrupts tumor angiogenesis in several experimental models and reveals exciting anti-tumor properties.

## RESULTS

### Design of peptides derived from the TSP-1 binding sequence of CD47

**Figure 1:**
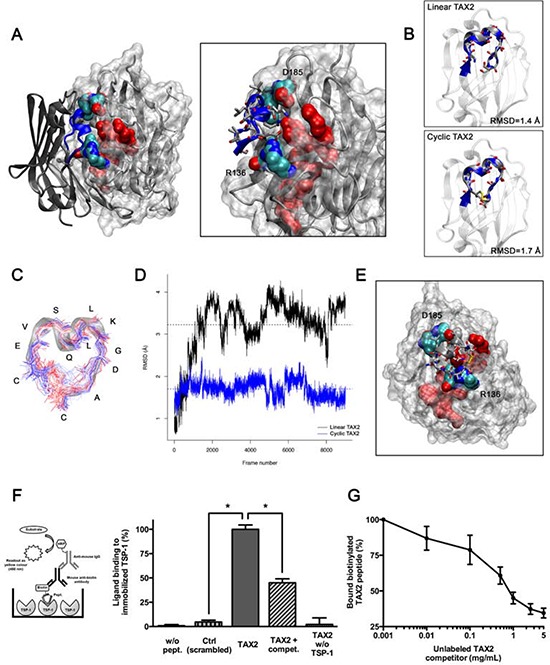
Identification and characterization of TAX2 peptides targeting the CD47-binding domain of TSP-1 **A.** *Left panel*. Result of rigid protein-protein molecular docking between an open conformation of TSP-1 CBD (MRMS = 2 Å; represented with a solvent-accessible surface on the protein) and the extracellular domain of CD47 (PDB ID code 2JJS, chain C). Solvent-accessible surface of RFYVVMWK interaction sequence on TSP-1 (*red*) is highlighted, as well as R136 and D185 residues allowing TSP-1 opening (*O is red, N is blue, C is cyan*). *Right panel* is a focus on the interaction region showing lateral chains of CD47 binding sequence IEVSQLLKGDAS (*blue*). **B.** Structural alignments of TAX2 predicted structures with CD47 extracellular domain. *Top panel* represents alignment of linear TAX2 (IEVSQLLKGDAS). *Bottom panel* represents alignment of the cyclic analogue (CEVSQLLKGDAC). Calculated RMSD values are indicated. **C.** Stable structure of TAX2 cyclopeptide as observed along 50 ns molecular dynamics simulation trajectory, highlighting that flexibility of the peptide is reduced to the disulfide bridge. **D.** RMSD from linear TAX2 (*black line*) and cyclic TAX2 (*blue line*) starting structures along the 50 ns simulation. Horizontal dotted line, mean. **E.** Result of molecular docking between minimized TAX2 cyclopeptide (represented as its secondary structure with lateral chains) and the open TSP-1 CBD. **F.** Thrombospondin from human platelets (5 μg/mL) was immobilized on ELISA microtiter plates and blocked with 1% (w/v) BSA. Biotinylated scrambled peptide (mentioned as Ctrl, 10 μg/mL), biotinylated TAX2 peptide (10 μg/mL) or non-labeled TAX2 competitor (1 mg/mL) were incubated in buffer containing 0.5% (w/v) BSA for 3 h at room temperature. Thrombospondin-bound biotin-labeled peptides were then quantified as described in Materials and Methods (see *left panel* scheme). Results are expressed as percentage of ligand bound to immobilized TSP-1 (mean ± SE, *n* = 3 independent experiments performed in triplicate, *t* test). **G.** Binding of biotinylated TAX2 peptide (10 μg/mL) to immobilized TSP-1 was assessed in the presence of increasing concentrations of unlabeled TAX2 competitor (0.001 to 5 mg/mL).

We recently demonstrated by using both normal mode analyses and energy minimizations that TSP-1 CBD can self-open, requiring disruption of an electrostatic scratch between R136 and D185 and leading to the solvent exposure of the CD47-interacting sequence RFYVVMWK [29] (Supplementary movie 1). These preliminary results allowed conducting molecular docking experiments between the extracellular domain of CD47 and an open conformation of the TSP-1 CBD (Fig. 1A). We thus identified the SQLLKGD receptor sequence as putatively involved in the TSP-1:CD47 interaction and proposed two CD47-derived dodecapeptides (called TAX2) that could bind TSP-1: the linear IEVSQLLKGDAS and its cyclic disulfide analogue CEVSQLLKGDAC. Structural alignments showed that PEP-FOLD predicted structures of both linear and cyclic TAX2 perfectly mimic the helical folding of the sequence in native CD47 (Fig. 1B). As shown in Figs. 1C and 1D, molecular dynamics experiments highlighted that the interacting sequence is less stretchy in the cyclic peptide. This correlates with increased stability over time and a conservation of the helical native conformation, suggesting an increased ability of cyclic TAX2 to bind TSP-1 CBD compared to the linear analogue. These data led us to favor the cyclic form of the peptide throughout our study. Docking experiments were also conducted between cyclic TAX2 and TSP-1 revealing that the CD47-derived peptide binds open TSP-1 CBD in a very similar way as the entire receptor (Fig. 1E). This suggests that the theoretically-predicted CD47-derived peptide might bind TSP-1 to antagonize TSP-1:CD47 interaction.

### CD47-derived TAX2 peptide directly binds TSP-1

We further investigated whether the CD47-derived peptide, proposed using molecular modeling, directly binds to TSP-1 using an ELISA binding assay with biotin-labeled peptides (Fig. 1F). The results highlighted that the TAX2 peptide binds to immobilized TSP-1 whereas the scrambled peptide does not. The specificity of the molecular interaction was controlled by competition with the corresponding non-biotinylated peptide. Competitive binding occurred in a dose-dependent fashion supporting this result (Fig. 1G). Consistently, the ability of TAX2 to bind TSP-1 was also confirmed by surface plasmon resonance and microscale thermophoresis (data not shown). These data demonstrate that the newly discovered CD47-derived peptide directly binds to TSP-1, and could prevent its endogenous interaction with CD47.

### TAX2 prevents angiogenesis both *in vitro* and *ex vivo*

**Figure 2:**
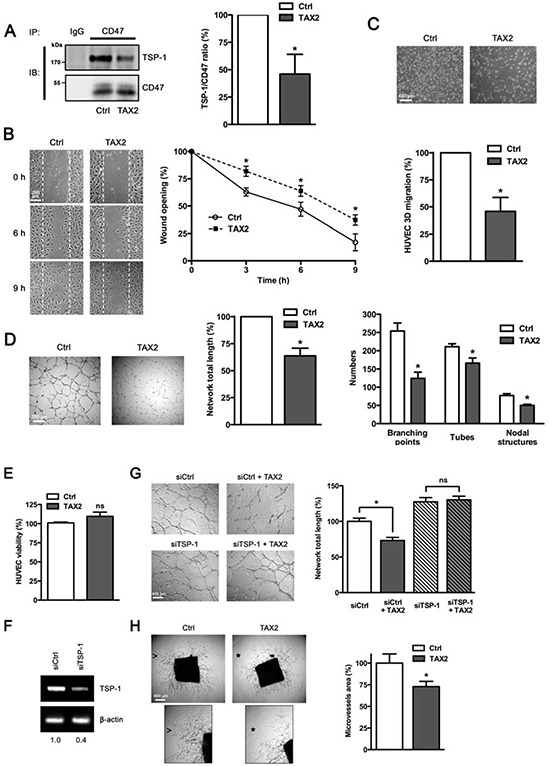
TAX2 peptide impairs endothelial cell migration and inhibits angiogenesis *in vitro* and *ex vivo* **A.** HUVECs were treated with TAX2 (100 μM) or scrambled peptide (Ctrl, 100 μM). CD47 was immunoprecipitated from total protein lysates using anti-CD47 (clone B6H12) and non-specific IgGs were used as a negative control. Immunocomplexes were then submitted to SDS-PAGE and immunoblotted (IB) using specific TSP-1 and CD47 antibodies. Histogram represents the quantification of co-immunoprecipitated TSP-1 relative to immunoprecipitated CD47. Results are expressed as percentage compared to control (*n* = 3 separate experiments, *t* test). **B.** HUVECs (1 × 10^5^ per well) were grown at confluence and then cell proliferation was inhibited by incubating with 10 μg/mL mitomycin C for 2 h. Migration assays were performed for 9 h with or without TAX2 peptide (100 μM), as described in Materials and Methods. Photographs (100×) were taken every 3 h. Graph represents the quantification of the wounded area. Each value is the mean of 3 independent experiments, each performed in quadruplicate. **C.** HUVECs (5 × 10^4^ per well) were plated into the upper compartment of a Transwell membrane and incubated for 12 h in EGM-2 in the presence of 100 μM TAX2 peptide. Hoechst-stained migrating cells attached on outside surface of the top chamber were photographed (20× objective) and counted. Results are expressed as percentage of migrated cells as compared to control. Each value represents the mean of 5 independent experiments, each performed in triplicate.*p < 0.05, compared to control; *t* test. **D.** HUVECs (5 × 10^4^) were cultured on matrigel layers in control conditions (Ctrl) or in the presence of TAX2. After 8 h incubation, HUVEC tube-like formation was observed on an inverted microscope (40×) and quantification of network total length was done using ImageJ software. Numeration of branching points, tubes and nodal structures was conducted using S.CORE system. Data are expressed as the mean ± SE from at least 3 independent experiments, each performed in triplicate. **E.** HUVEC viability was assessed and expressed as percent compared to control treatment (*t* test, ns, not significant). **F.** Total RNAs were purified from HUVECs transfected with non-silencing siRNA (siCtrl) or siRNA targeting TSP-1 (siTSP-1). The transcriptional level of TSP-1 was assessed by RT-PCR and normalized to β-actin. Numbers under the gel indicate the fold change as compared to siCtrl cells used as reference. **G.** Tube formation assay was performed as described above. HUVECs transfected with siRNA sequences (siCtrl and siTSP-1) were allowed to form tubes on matrigel during 8 h in presence or absence of TAX2 (*, *p* < 0.05; ns, not significant; *t* test). **H.** Aortic ring assays were performed as described in the experimental procedure. After 6 days incubation in the presence of 100 μM TAX2 or control peptide (Ctrl), images were taken with an inverted microscope (40×) and microvessel area was quantified using ImageJ software. Insets from control conditions (*arrowhead*) and TAX2 treatment (*star*) highlight that the microvessel sprouting in matrigel was inhibited by TAX2 (zoomed in 200%). *, *p* < 0.05, compared to control (*t* test).

As TSP-1 or TSP-1-derived peptides that bind to CD47 have been extensively reported to inhibit angiogenesis [12, 25], we investigated whether TAX2 treatment could rescue angiogenesis using *in vitro* and *ex vivo* models. First co-immunoprecipitation assays were carried out to confirm that TAX2 specifically prevents TSP-1 binding to CD47 in human primary endothelial cells (Fig. 2A). However, TAX2 does not affect TSP-1 binding to soluble partners such as FGF-2 and VEGF nor its ligation to other membrane receptors such as β1 integrin and LRP-1 (Fig. S1) [30, 31]. As endothelial cell migration is critical to form blood vessels [32], a wound-healing assay was then conducted to quantify endothelial cell migration under TAX2 treatment. Contrary to our initial expectation, a reduced migration rate was observed when endothelial cells were treated by the CD47-derived peptide compared to control (Fig. 2B). Endothelial cell migration was also assessed using a Boyden chamber assay (Fig. 2C). TAX2 inhibited endothelial cell migration by 50% in this 3D-like system, consistent with the results obtained with the 2D wound-healing model. A dose-response assay showed that the optimal inhibition is reached at a 100 μM concentration of TAX2 (data not shown). The unexpected inhibition of endothelial cell migration induced by TAX2 suggests that the newly characterized TSP-1:CD47 antagonist could act as prospective anti-angiogenic agent. To investigate the functional consequences of the CD47-derived peptide on microvascular network formation, we first conducted an *in vitro* assay allowing lumen formation by HUVECs on matrigel. As shown in Fig. 2D (left and middle panels), TAX2 inhibited the formation of a pseudo-tube network by 40% as assessed by quantification of total network length. Accordingly, the formation of branching points, capillary tubes and nodal structures was also decreased under TAX2 treatment (Fig. 2D, right panel). The decrease in endothelial cells ability to assemble in well-formed tube networks cannot be attributed to any cytotoxic effect of TAX2 (Fig. 2E). RNA interference-mediated knock-down of TSP-1 gene expression confirmed that TAX2 effects on lumen formation are specifically mediated by TSP-1 targeting, as they were wholly abolished under TSP-1 gene silencing (Figs. 2F and 2G). To confirm the results observed with this angiogenesis cell-based assay, TAX2 properties were also evaluated in a more physiologically relevant setting using a mouse aortic ring assay [33]. As shown in Fig. 2H, microvessel sprouting from the aorta was markedly inhibited by the CD47-derived peptide, supporting its unpredicted but exciting anti-angiogenic effect.

### TAX2 anti-angiogenic properties are mediated by TSP-1 binding to CD36

**Figure 3:**
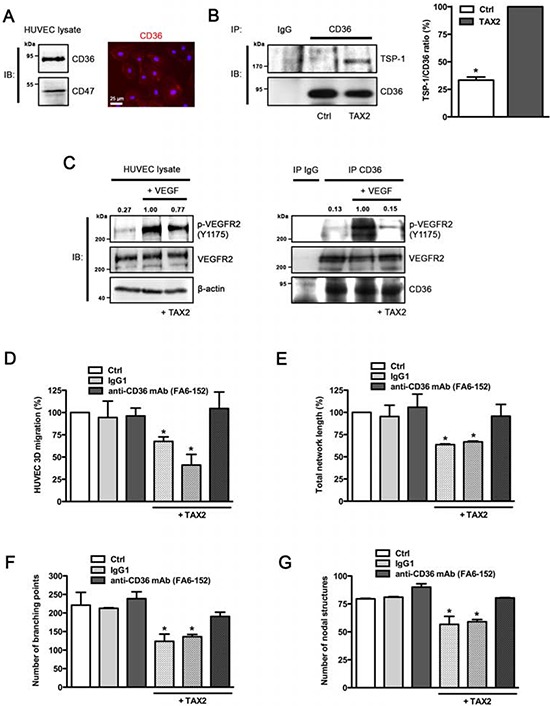
Anti-angiogenic properties of TAX2 are mediated by TSP-1 binding to CD36 **A.** Expression of CD36 in HUVECs was assessed by immunoblotting (*left panel)* and immunofluorescence staining (*right panel, red*). Nuclei were counterstained with DAPI (*blue*). **B.** HUVECs were incubated with 100 μM cyclic TAX2 or in control conditions for 24 h. Then, CD36-containing complexes were co-immunoprecipitated from whole cell extracts (non-specific IgGs were used as a negative control for immunoprecipitation). Immunocomplexes were submitted to SDS-PAGE and immunoblotted (IB) by using specific antibodies for TSP-1 and CD36. The presence of TSP-1 in immunocomplexes was quantified by densitometric analysis relative to immunoprecipitated CD36. **C.** VEGFR2 phosphorylation induced by VEGF in the presence of TAX2. After overnight serum starvation, HUVECs were pre-treated with 100 μM TAX2 or control for 12 h, then incubated with 30 ng/mL VEGF for 5 min. Whole cell lysates (*left panel*) or immunoprecipitated CD36-containing complexes (*right panel*) were submitted to SDS-PAGE and immunoblotted (IB) using anti-p-VEGFR2 (Y1175), anti-VEGFR2 and anti-β-actin or anti-CD36 antibodies. Upper numbers indicate the value of pVEGFR2/VEGFR2 ratio by densitometry (*n* = 3). **D.** Transwell migration assay was performed as described for Fig. 2C. HUVECs were allowed to migrate for 12 h under TAX2 or control treatment (100 μM) and with/without anti-CD36 blocking antibody (10 μg/mL). Non-reactive IgG1 serves as control for anti-CD36 treatment. Data are expressed as the mean ± SE (*n* = 3; *p < 0.05, ANOVA). **E-G.** HUVECs were incubated for 12 h in endothelial cell growth medium with 100 μM TAX2 or control peptide and with/without anti-CD36 blocking antibody (non-specific IgG1 serves as control). Tube formation assay was performed as described for Fig. 2D. Quantification of total tube-like network length was realized using ImageJ software **E.** Branching points **F.** and nodal structures **G.** were numbered using the S.CORE web-based system. Data are expressed as the mean ± SE (*n* = 3; *p < 0.05, ANOVA).

The anti-angiogenic properties of TAX2 were unexpected since TSP-1 binding to CD47 was mainly reported to promote arterial vasoconstriction and to inhibit VEGFR2 signaling [13, 14]. Considering that CD36 is also implicated in angiogenesis inhibition in response to soluble TSP-1 [11, 13], we investigated whether the anti-angiogenic effects of TAX2 could be mediated by CD36. As expected, we found that CD36 is highly expressed in HUVECs (Fig. 3A). The amount of TSP-1 co-immunoprecipitated with CD36 increased by 3-fold in the presence of TAX2 (Fig. 3B), indicating that TAX2 enhances TSP-1 binding to CD36 in HUVECs. Since VEGFR2 can associate as a dimer with a CD36/β1 integrin complex to form a cluster of membrane receptors regulating angiogenesis [13, 34], we further investigated the ability of TAX2 to inhibit VEGF-induced activation of VEGFR2. As shown in Fig. 3C (left panel), TAX2 partially reverses VEGF-induced VEGFR2 phosphorylation in HUVECs. This effect may be due to the association of activated VEGFR2 to a CD36-containing complex, as revealed by CD36 co-immunoprecipitation assays (Fig. 3C, right panel). Consistent with these data concerning VEGFR2 phosphorylation state, TAX2 treatment also decreases cGMP production by endothelial cells after NO stimulation by around 25% (Fig. S2). Furthermore, the ability of TAX2 to prevent endothelial cell migration (Fig. 3D) and formation of capillary structures (Figs. 3E to 3G) is wholly reversed in the presence of a CD36 blocking antibody. Altogether, these data demonstrate that TAX2 promotes TSP-1 binding to CD36-containing complexes, leading to disruption of VEGFR2 signaling.

### TAX2 induces extensive melanoma tumor necrosis *in vivo*

**Figure 4:**
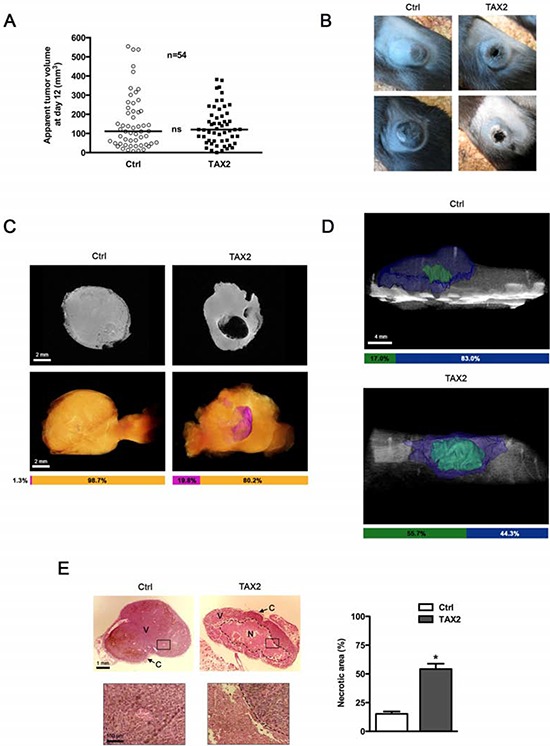
TAX2 induces central tumor necrosis in a mouse melanoma allograft model **A.** 2.5 × 10^5^ melanoma B16F1 cells were subcutaneously injected into the left side of C57BL/6 mice. Control (Ctrl, open circle) or TAX2 (filled box) peptide (10 mg/kg) was intraperitoneally injected at days 3, 5 and 7. Tumor volume was measured with a caliper as described in Materials and Methods. Graph represents tumor volume measurements at day 12 after melanoma cell injection. *Horizontal lines*, median (*n* = 54, *Mann-Whitney* test). **B.** Tumor photographs at day 20 from 2 mice treated with scrambled (Ctrl, *left panel*) or TAX2 (*right panel*) peptide. **C.** *Ex vivo* MRI analysis of isolated tumors at day 12 after tumor challenge. *Top panel*: 2D virtual slice at the center of tumor volume. *Bottom panel*: Tumor 3D reconstruction (*orange*: tumor, *purple*: necrotic area). Horizontal bars show the respective proportions of necrotic area and viable tumor tissue as quantified using Amira 5.4.3 software (representative of *n* = 10). **D.** μCT analysis of mice at day 14 after tumor challenge: tri-dimensional reconstruction after segmentation of melanoma tumor (*blue*) and the associated necrotic core (*green*) using Amira 5.4.3 software. Horizontal bars show distribution of necrotic and viable parts within tumor volume (representative of *n* = 10). **E.** HPS staining of tumor sections showing tumor necrosis (*dotted line*). Insets (*correspond to black rectangle in tumor sections, zoomed* ×100) highlight the dense tumor tissue and its vascularization in viable areas (V) and cellular debris in the necrotic area (N) Tumor periphery is surrounded by a fibrotic capsule region (C). Histogram: quantification of the necrotic area relative to total tumor surface on each HPS-stained histological section using ImageJ software. Data are expressed as the mean ± SE (*p < 0.05, *t* test).

To investigate whether TAX2 could prevent tumor development by inhibiting angiogenesis, B16F1 melanoma cells were subcutaneously inoculated in the left flank of C57BL/6 mice prior to three intraperitoneal administrations of TAX2 at days 3, 5 and 7. No significant modification in the measured median tumor volume was found for TAX2-treated mice compared to control mice during early stages of tumor development (Fig. 4A). However, mice treated with the CD47-derived peptide exhibited a substantial tumor necrosis at day 20 after tumor challenge (Fig. 4B). Tumor necrosis was visualized upstream through a multimodal imaging approach combining magnetic resonance imaging (MRI) analysis of isolated tumors (Fig. 4C) and quantitative micro-computed tomography (μCT) imaging of mice (Fig. 4D). The manual measurement of tumor size did not give rise to any differences between TAX2-treated mice and control mice (Fig. 4A). However, quantitative imaging highlighted that the CD47-derived peptide could decrease tumor proper volume. Indeed, both MRI and μCT highlighted a large central necrotic area inside tumor masses from TAX2-treated mice whereas such necrotic cores remained much more restricted for control mice (Figs. 4C and 4D, see volume quantification bars under the images). Furthermore, these imaging observations were correlated to histopathological HPS staining showing that the necrotic area in TAX2-treated mice was increased by about 3-fold compared to control mice (Fig. 4E). Interestingly, neither μCT imaging of healthy organs nor mice dissections highlighted any sign of thrombosis, hemorrhage or embolism (data not shown).

### TAX2 peptide substantially disturbs melanoma tumor vascularization *in vivo*

**Figure 5:**
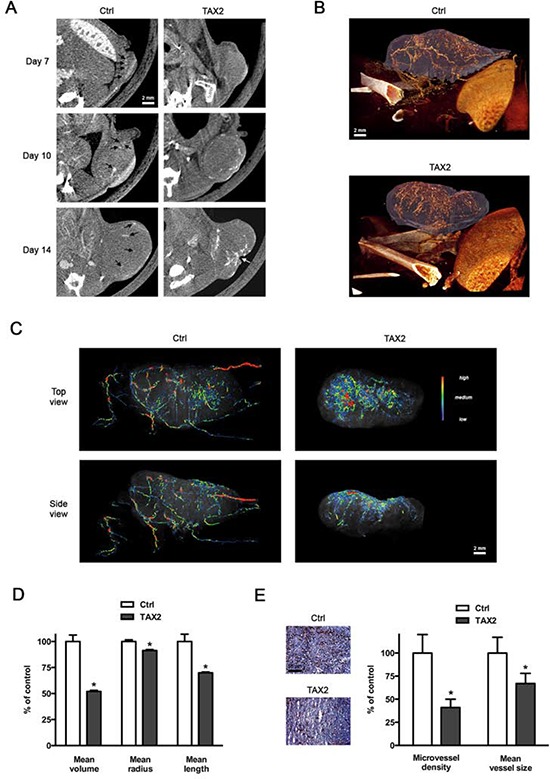
TAX2 disturbs tumor angiogenesis **A.** 2.5 × 10^5^ melanoma B16F1 cells were subcutaneously injected into the left side of C57BL/6 mice, then intraperitoneal administrations of scrambled (Ctrl, *left panel*) or TAX2 (*right panel*) peptide (10 mg/kg) were performed at days 3, 5 and 7. Intravenous injections of an alkaline earth-based contrast agent (100 μL) were performed before mice imaging at days 7, 10 and 14 to allow a longitudinal follow-up of tumor angiography through μCT analysis. *Black arrows* illustrate contrast enhancement of intratumoral blood vessels; *white arrow* indicates tumor necrosis (representative of *n* = 5). **B.** Tridimensional reconstructions of tumors at day 14. Melanoma tumor (*purple*) and the associated vascular network (*orange*) were segmented using Amira 5.4.3 software. **C.** Color-coded representation of tumor-associated blood vessel network depending on structure thickness (Amira 5.4.3 software). Thin structures are represented in blue, which changes to green and red when the vessel diameter increases. **D.** Quantification of tumor-associated blood vessel mean length, volume and radius using Amira 5.4.3 software. Data are expressed as the mean ± SE (*p < 0.05, *t* test). **E.** CD31 immunostaining of tumor frozen sections. Quantification was done using ImageJ software, and results are expressed as a percentage of control MVD and blood vessel mean size (mean ± SE, *n* = 6, *t* test).

To determine whether tumor necrosis could be due to TAX2-induced inhibition of tumor angiogenesis, we performed a longitudinal follow-up of melanoma allograft angiography using μCT monitoring after intravenous injection of an alkaline earth-based nanoparticulate contrast agent [35]. While vascular structures were easily visualized within tumors of control mice at day 7 after tumor challenge, blood vessels were undetectable within tumors of TAX2-treated mice (Fig. 5A, top panel). Intratumoral vascularization remained prominent within growing tumors of control mice, while it appeared restricted to tumor periphery under TAX2 treatment at day 10 (Fig. 5A, middle panel). At day 14, a central vascular network within tumors from TAX2-treated mice was still lacking and a wide hemorrhagic area corresponding to the previously described necrotic zone was observed (Fig. 5A, bottom panel). Tridimensional reconstructions 14 days after tumor challenge revealed a mature peritumoral and intratumoral vascular network in control mice (Fig. 5B, upper panel). In contrast, after TAX2 treatment, tumor vasculature appeared highly anarchic and discontinuous, only at the tumor surface, surrounding a central hemorrhagic area (Fig. 5B, lower panel). Segmentation and quantification of the tumor-associated vascular network revealed that TAX2 induces a 2-fold reduction in blood vessel mean volume, as the treatment significantly decreased both the length and diameter of blood vessels (Fig. 5C, 5D and Supplementary movie 2). In accordance with these results, CD31 immunostaining of tumor cryosections reveals a 60% diminution in intratumoral MVD associated to a diminution in vessel size (Fig. 5E). In light of these observations and in consistence with results from *in vitro* and *ex vivo* assays, we established using μCT longitudinal analysis that TAX2 acts as a new anti-angiogenic agent.

### TAX2 treatment delays growth of human pancreatic carcinoma xenografts

**Figure 6:**
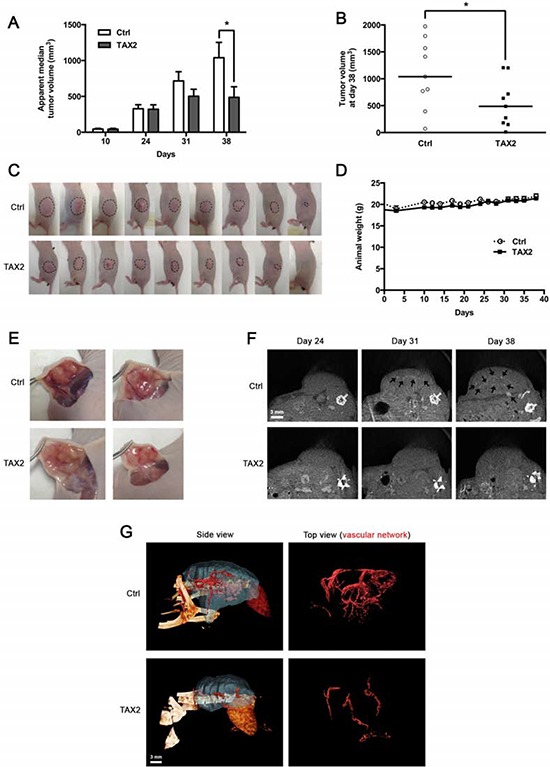
TAX2 inhibits growth of human pancreatic carcinoma tumor xenografts **A.** 3 × 10^6^ pancreatic carcinoma MIA PaCa-2 cells were subcutaneously injected into the left side of BALB/C *nu/nu* mice. Control (Ctrl) or TAX2 peptide (10 mg/kg) was intraperitoneally injected 3 times a week for 4 weeks starting at day 10 after tumor challenge. Tumor volume was measured with a caliper as described in Materials and Methods. Histogram represents the evolution of tumor xenograft volume. Data are expressed as apparent median tumor volume ± SE (*p < 0.05; *Mann-Whitney* test; *n* = 15 from days 10 to 24, *n* = 11 at day 31, *n* = 9 at day 38). **B.** Tumor volume measurements at day 38 after pancreatic tumor cells injection. *Horizontal lines*, median (*n* = 9; *Mann-Whitney* test; *p < 0.05). **C.** Tumor photographs at day 38 after pancreatic tumor cells injection. Dotted lines show tumor contours. **D.** Mouse mean weight evolution for control (Ctrl, *open circle*) and TAX2-treated (*filled box*) groups. **E.** Tumor dissection photographs at day 38 for 2 mice from control group (Ctrl, *top panel*) or TAX2-treated group (*bottom panel*). **F.** μCT imaging of mice at days 24, 31 and 38 after intravenous injection of an alkaline earth-based contrast agent (100 μL) allowing tumor angiography analysis. *Black arrows* illustrate contrast enhancement of intratumoral blood vessels (representative of *n* = 5). **G.** Tridimensional reconstructions of tumors at day 38. Human pancreatic carcinoma xenograft (*blue*) and the associated vascular network (*red*) were segmented using Amira 5.4.3 software.

While significant defects in vascularization and extensive tumor necrosis were observed in the syngeneic subcutaneous melanoma model, there was no change regarding apparent tumor volume (Fig. 4A). As this conflicting result might be explained by the notorious invasiveness of the B16 model, we decided to extend the results in a less aggressive model of human pancreatic carcinoma xenograft. Intraperitoneal administrations of TAX2 after tumor pre-establishment induced a strong delay in MIA PaCa-2 subcutaneous xenografts growth (Fig. 6A); resulting in a 2-fold decrease of tumor volume at day 38 after cells inoculation (Figs. 6B and 6C) while mice did not exhibit any other clinical sign or weight loss (Fig. 6D). Mouse dissections disclosed a correlation between reduced tumor volume and decreased tumor vasculature under TAX2 treatment (Fig. 6E). Analysis of tumor angiography by μCT revealed that intratumoral blood vessels were undetectable within subcutaneous xenograft at day 24 after tumor challenge (Fig. 6F, *left panel*), when no differences in measured tumor volume were observed at this stage (Fig. 6A). Longitudinal follow-up until day 38 showed prominent intratumoral blood vessels within growing tumors of control mice, while decreased tumor growth was accompanied by a striking alteration of tumor vascularization under TAX2 treatment (Fig. 6F, *middle and right panels*). Tridimensional reconstructions at day 38 after tumor challenge confirmed that TAX2 administrations strongly inhibit tumor-associated vascular network (Fig. 6G). Altogether, these data presents TAX2 peptide as a new anti-angiogenic agent suppressing tumor vasculature.

## DISCUSSION

In the present study, we used molecular docking experiments to identify an innovative dodecapeptide sequence derived from the cell surface receptor CD47 that antagonizes TSP-1:CD47 interaction. We demonstrate that the *in silico*-designed peptide, called TAX2, exhibits unexpected anti-angiogenic properties *in vitro* and *ex vivo* and induces extensive melanoma tumor necrosis *in vivo* by inhibiting tumor angiogenesis. Moreover, TAX2 peptide also significantly inhibits tumor growth in a human pancreatic carcinoma xenograft model. These results were obtained by means of a large number of animals and samples using innovative multimodal and multi-scale imaging approaches that were supported by histopathological staining.

The anti-angiogenic properties of TAX2 were quite surprising and unexpected since TSP-1 binding to CD47 has been mainly reported to promote vasoconstriction and limitation of angiogenesis. Indeed, by using TSP1-derived peptides and recombinant fragments of TSP-1, Isenberg and collaborators demonstrated that the anti-angiogenic activity of TSP-1 is mainly mediated *via* binding to CD47, resulting in inhibition of NO-induced activation of guanylate cyclase [25]. Besides the modulation of cyclic nucleotide levels, TSP-1 CBD ligation to CD47 was recently reported to disrupt the constitutive association of CD47 with VEGFR2 in endothelial cells, resulting in inhibition of VEGFR2 activation and downstream signaling [14].

Here, we do not dispute the fact that TSP-1 is capable of inhibiting angiogenesis by different routes. However, we are convinced that the selective inhibition of TSP-1 interaction with CD47 can induce a previously unpredicted overall anti-cancer response, to be explored and deciphered. Although the anti-angiogenic and anti-tumor properties of TAX2 were initially not predicted, they are nevertheless consistent with results previously reported by Maxhimer and collaborators [36] who emphasized the therapeutic value of blocking TSP1:CD47 interaction by using the same experimental *in vivo* melanoma model. This study also suggests that favorable combination of TAX2 with radiotherapy would be promising to improve therapeutic response and treatment tolerance. Interestingly, the effects of TAX2 appear to be restricted to the tumor-associated environment overexpressing TSP-1, as no signs of thrombosis, hemorrhage or embolism were observed in healthy organs in both syngeneic melanoma and human pancreatic carcinoma models. We are convinced that the unexpected effects of TAX2 could be attributable to an intense matrix remodeling occurring within tumor-associated microenvironment, in which matricellular proteins, including TSP-1 and CD47 ligands, may reach bioavailable concentrations that overcome physiological levels. By disregarding the main changes in the molecular balances between the respective ligands occurring in the highly reactive tumor microenvironment, we impede our thinking and limit our ability to improve existing treatments. TSP-1 was indeed reported to be over-expressed in tumor stroma [37], and its contribution through CD47 ligation remains controversial in this context due to the pleiotropic nature of TSP-1 and its ability to induce opposite effects in different host organ environments [16, 23]. In myeloma, the potential importance of targeting CD47-TSP-1 axis has already been highlighted [38]. Consistent with our data, CD47 was found to be elevated in clinical melanoma samples and delivery of siRNA targeting CD47 was recently reported to effectively inhibit melanoma tumor growth and lung metastasis [39]. Moreover, recent data pointed out that CD47 mRNA expression in patients are correlated with decreased survival in several cancer types and antibody blockade of CD47 yielded promising results in mice bearing human xenografts reducing tumor growth as well as metastasis [40]. Strategies based on CD47 blockade are also currently under development giving promising results in multiple pancreatic cancer preclinical models [41]. However, caution must be raised when comparing TAX2 biological effects to previous results obtained by using blocking CD47 antibodies. TAX2 treatment only targets TSP-1 and may not affect CD47 association with other ligands such as integrins or SIRP-α as the structures involved are not redundant with those implicated in the molecular complex interface (Fig. S3) [4, 42]. Moreover, anti-CD47 IgG may also induce Fc-mediated effects such as antibody-dependent phagocytosis or cytotoxicity. Using TAX2 provides new opportunities to bypass the problem of existing molecules blindly targeting CD47 towards its ligands that may have major effects on tumorigenesis.

**Figure 7:**
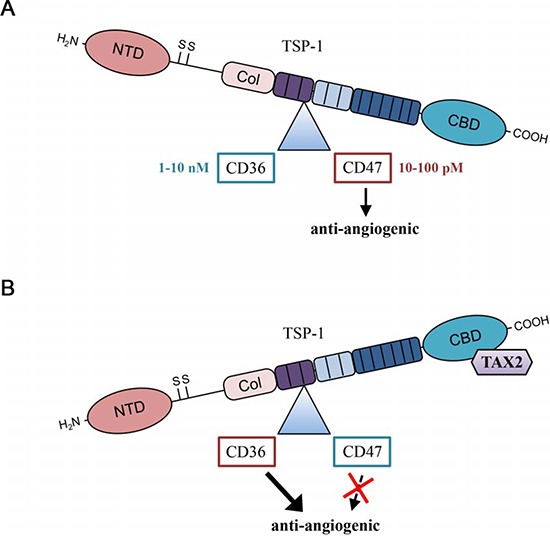
TAX2 induces a shift in TSP-1 binding from CD47 to CD36 **A.** In physiological conditions, most of TSP-1-related effects on vascular function are mediated by TSP-1 carboxy terminal domain interaction with CD47 receptor. **B.** Under TAX2 treatment preventing TSP-1:CD47 interaction, binding of the 3TSR module of TSP-1 to CD36 receptor is promoted, thus leading to an overall anti-angiogenic response (*NTD, N-terminal domain; Col, procollagen-homology domain; CBD, carboxy terminal cell binding domain*).

In addition to the putative effects that may be due to CD47 association with its co-receptors, TAX2-bound TSP-1 also remains able to bind a wide variety of ligands through the other domains of the multi-modular protein. Among these domains, a central region of TSP-1 called 3TSR (three TSP-1 type 1 repeats) binds to the CD36 membrane receptor, also leading to inhibition of angiogenesis. Indeed, CD36 ligation by TSP-1 is known to induce an anti-angiogenic response by promoting CD36 association with β1 integrin and VEGFR2 dimer in a tripartite complex, resulting in decreased VEGFR2 phosphorylation after VEGF stimulation [13]. Physiological plasmatic TSP-1 concentration at around 100 pM favors TSP-1 ligation to CD47, as the binding affinity is higher compared to CD36 binding [43]. To explain our apparently contradictory results, we hypothesized that TAX2 peptide inhibiting TSP-1: CD47 interaction may increase the amount of bioavailable soluble TSP-1 that is free from CD47 engagement, thus stimulating TSP-1 3TSR binding to CD36 and resulting to an anti-angiogenic response. Using CD36 co-immunoprecipitation experiments, VEGFR2 phosphorylation assays and validated CD36 blocking antibodies, we established that TAX2 induced a shift in TSP-1 binding from CD47 to CD36 (Fig. 7). Accordingly, we propose that TAX2 prevents VEGFR2 activation by VEGF and inhibits subsequent NO-cGMP signaling, leading to inhibition of endothelial cell migration and abrogation of the formation of capillary-like structures. Furthermore, we found using TSP-1 ELISA assay that soluble TSP-1 reaches nanomolar concentrations compatible with CD36 activation in our endothelial cell environment (data not shown) [25]. CD36 has already been considered as a target of interest in the development of TSP-1-based anti-angiogenic therapies. Dawson and colleagues first described in 1999 a modified TSP-1 type 1 repeats-derived nonapeptide (since referenced as ABT-510) exhibiting strong anti-angiogenic and anti-neoplasic activities [44]. By modulating endothelial cell proliferation and migration as well as apoptosis in CD36-expressing cancer cells [45], ABT-510 targets both tumor cells and the associated tumor vascular network. Indeed, significant effects of ABT-510 on tumor growth and vascularization, especially microvessel density, were reported *in vivo* in many cancers including glioma [46], ovarian carcinoma [45], Lewis lung carcinoma [47] and bladder cancer [48]. Several phase I and phase II clinical trials have been conducted with ABT-510 since 2005. These trials have sometimes led to intricate and nuanced outcomes mainly due to a lack of clinical activity when used as a single agent with frequently observed severe adverse events such as thrombosis and pulmonary embolism [49, 50]. By concomitantly inhibiting TSP-1 binding to CD47 and enhancing its ligation to CD36, TAX2 may induce a weaker anti-angiogenic response than the one resulting from 3TSR stimulation of CD36, which might therefore significantly reduce the risk of thrombosis.

In the subcutaneous B16 melanoma model, systemic administrations of TAX2 peptide during the early stages of tumor growth induces a delayed but extensive tumor necrosis, as evidenced by MRI and μCT. Regarding the results obtained in our *in vitro* and *ex vivo* models, we assume that TAX2-related effects may mostly be attributed to stromal cells in the TSP-1-enriched tumor stroma, especially through endothelial cell targeting. Analysis of tumor angiography highlighted that tumor-associated vascular network appears significantly disrupted and unstructured as a delayed consequence of TAX2 treatment, which was consistent with the results obtained from the *in vitro* morphogenesis assay.

Given the fact that TAX2 anti-cancer effects were reproduced in immunodeficient BALB/C *nu/nu* athymic mice, the eventual immunological contribution that may be involved in TAX2-derived anti-tumor effects could be de-emphasized. In the less aggressive MIA PaCa-2 pancreatic carcinoma xenograft model, systemic administrations of TAX2 peptide decrease growth of pre-established subcutaneous tumors 2-fold by considerably altering tumor-associated vasculature. Consistent with our proposed mechanism of action that involves an enhanced activation of the TSP-1/CD36 axis under TAX2 treatment, these experimental data are also coherent with previous work underlying the anti-angiogenic and anti-tumor potential of the CD36-binding 3TSR module of TSP-1 in human pancreatic cancer models [51, 52]. Indeed, the relevance of targeting VEGFR signaling in pancreatic cancer has also been demonstrated [53]. An obvious approach for VEGF inhibition is to decrease the availability of ligand (VEGF), as demonstrated by bevacizumab, a humanized murine anti-human VEGF-A monoclonal antibody that has been used in the treatment of pancreatic cancer together with other chemotherapy agents [54, 55]. In the MIA PaCa-2 xenograft model, our results show that TAX2 administrations led to higher inhibition rates compared to 5 mg/kg bevacizumab intraperitoneally injected twice weekly [56], suggesting that TAX2 peptide may be useful upon development of tumor resistance against bevacizumab and other conventional anti-angiogenic therapies. Indeed, recent studies showed that anti-VEGF treatment could make tumor more aggressive and metastatic [57]. While the mechanisms and mediators of this phenomenon are not yet all understood, emerging evidence indicates that angiogenesis targeting agents may induce a “metastatic switch” as a result of hypoxia in certain experimental conditions [58]. Interestingly, TAX2 did not increase metastasis formation considering an experimental model of lung metastasis using invasive B16F10 melanoma cells (data not shown), confirming that using TAX2 alone or in combination with other chemotherapeutic agents may be highly relevant for future therapeutic approaches.

Overall, we have developed and characterized an innovative and non-obvious molecule that targets TSP-1 at the CD47 binding site and exhibits unpredicted but exciting overall anti-cancer properties *in vivo*. These data emphasize the therapeutic value of blocking TSP1:CD47 interaction for inhibiting tumor vascular network. This challenges the current dogma on TSP-1 and provides new conceptual insight that could supply innovative therapeutic approaches against malignant diseases.

## MATERIALS AND METHODS

### Cell culture, reagents and antibodies

B16F1 cancer cell line was obtained from the American Type Culture Collection (ATCC) and maintained in RPMI-1640 medium (Gibco, Life Technologies, Saint-Aubin, France) supplemented with 10% fetal bovine serum (FBS). CD47 expression in B16F1 cells was checked by immunoblot. MIA PaCa-2 cell line was obtained from ATCC and maintained in Dulbecco's Modified Eagle's Medium (Gibco) supplemented with 10% FBS and 2.5% horse serum. HUVECs pre-screened for angiogenesis and vascular cell health markers (including eNOS and VEGF-R2) were purchased from Lonza Clonetics (Basel, Switzerland) and maintained in complete endothelial growth medium (EGM-2, Lonza) at early passages (up to 4). All cell lines were authenticated before experiments according to the provider's protocol. Cell viability and cell proliferation were assessed using UptiBlue Viable Cell Counting Kit (Uptima, Interchim, France). Thrombospondin from human platelets and human recombinant VEGF-165 were purchased from Calbiochem (Merck Millipore, Darmstadt, Germany) and PromoKine (PromoCell, Heidelberg, Germany), respectively. Anti-biotin antibody (#MB-9100) was purchased from Vector Laboratories (Abcys, Les Ulis, France). Anti-CD47 (clone B6H12, #556044) was from BD Pharmingen (Le Pont-de-Claix, France). Anti-TSP-1 (clone A6.1, #ab1823) and anti-β1 integrin (clone BV7, #ab7168) were from Abcam (Paris, France). Anti-LRP light chain (clone 5A6, #438192) and heavy chain (clone 8G1, #438190) were from Calbiochem. Anti-CD36 (clone FA6–152, #sc-52645), anti-β-actin (#sc-1616), anti-p-VEGFR2 (Y1175, #CC1028), anti-VEGFR2 (#CC1020), anti-FGF-2 (clone C-18, #sc-1360), anti-VEGF (clone C-1, #sc-7269) and normal mouse IgG (#sc-2025, used as a control for immunoprecipitation) were from Santa Cruz Biotechnology (Tebu-bio, Le Perray-en-Yvelines, France). HRP-linked anti-mouse IgG (#7076S) and anti-rabbit IgG (#7074S) used as secondary antibodies for Western blots were purchased from Cell Signaling Technology (Ozyme, Saint-Quentin-en-Yvelines). HRP-linked anti-goat IgG (#A5420), DEA-NONOate (2-(N, N-Diethylamino)-diazenolate 2-oxide sodium salt hydrate) and IBMX (3-isobutyl-1-methylxanthine) were obtained from Sigma-Aldrich (Saint-Quentin Fallavier, France). Anti-CD31 antibody (clone MEC 13.3) used for immunohistochemistry was from BD Pharmingen (#557355) and biotinylated anti-rat IgG (#BA-4001) used as secondary antibody was Vector Laboratories (Abcys). Secondary Alexa Fluor 568-conjugated antibodies and Prolong Gold antifade reagent with DAPI were from Molecular Probes (Invitrogen, Cergy-Pontoise, France).

### Molecular docking and simulations

Rigid protein-protein docking of CD47 (PDB ID code 2JJS [42], chain C) was performed against an open conformation of TSP-1 CBD, as previously determined by normal mode analysis [29] using the GRAMM-X web server v1.2.0. A refinement of the resulting orientations was further performed using RosettaDock (Rosetta 3.4 software). The structure of the CD47-derived peptide (TAX2) was predicted using the PEP-FOLD online resource in order to verify that the chosen sequence mimics the native helix conformation. Predicted structures of this linear peptide and of its cyclic disulfide analogue (manually edited and minimized) were compared to the corresponding X-ray structure in 2JJS using VMD software. Molecular dynamics simulations in explicit solvent (TIP3P) were performed on both peptides using NAMD software together with the CHARMM27 force field, in the NPT ensemble at 293 K - 1 atm, with a cut-off of 11 Å for long-range interactions. The relative flexibility of both peptides was assessed by means of Root Mean Square Deviation (RMSD) calculations. Molecular docking between the minimized cyclic peptide and the TSP-1 open conformation was done using Autodock 4.2 software (population size of 200, 10 × 10^6^ energy evaluations, 250 LA-GS runs). Figures and graphs were generated using VMD and R software, respectively.

### Designed peptides

The CD47-derived linear dodecapeptide (IEVSQLLKGDAS), its cyclic disulfide analogue (CEVSQLLKGDAC), the scrambled dodecapeptide used as a control (LSVDESKAQGIL) as well as the corresponding biotinylated peptides were synthesized and purified by Genecust (Dudelange, Luxembourg). Each peptide was controlled following synthesis for composition and purity through electrospray ionization-mass spectrometry (ESI-MS) and HPLC. Disulfide bridge cyclisation was ensured by Raman spectroscopy. Peptides were solubilized in the appropriate medium for *in vitro* and *ex vivo* assays, or in normal saline solution (0.9% (w/v) NaCl) for *in vivo* experiments.

### Binding of biotin-labeled peptides to immobilized TSP-1

Microtiter plates (Nunc, ThermoFisher Scientific, Courtaboeuf, France) were coated overnight at 4°C with 0.5 μg TSP-1 in sodium carbonate-bicarbonate (BupH) buffer (Thermo Fisher Scientific). Plates were washed with PBS 0.1% BSA (Sigma-Aldrich) and non-specific sites were saturated during 30 min using 1% (w/v) BSA in BupH buffer. Biotin-labeled peptide (10 μg/mL, determined after dose-response assay from 0.01 to 100 μg/mL) was added in buffer containing 0.5% (w/v) BSA and incubated for 3 h at room temperature. Non-labeled peptide (0.001 to 5 mg/mL) was used as selective competitor. Plates were washed with PBS containing 0.1% (w/v) BSA and treated for 30 min with mouse anti-biotin antibody. Bound antibody was quantified using the mouse IgG Elite Vectastain ABC kit (Vector Laboratories) followed by incubation with 1-Step Turbo TMB substrate as a chromogen (ThermoFisher Scientific). The reaction was stopped with 0.5 M H_2_SO_4_ and absorbance was read at 450 nm.

### Protein extraction and Western blot analysis

Cells were harvested on monolayers, washed with ice-cold PBS and then solubilized in lysis buffer (50 mM HEPES, pH 7.6, 100 mM NaCl, 20 mM EDTA, 1% (v/v) Triton X-100, 0.5 mM PMSF) supplemented with Halt protease and phosphatase inhibitor cocktail (Thermo Fisher Scientific). The protein concentration was quantified using the BCA Assay kit (Uptima). Lysates were loaded on 10% polyacrylamide gels and separated by SDS-PAGE. Proteins were transferred to nitrocellulose membranes (Amersham Biosciences), blocked with dry milk solution (5% w/v) or BSA (5% w/v) for phospho-proteins and immunoblotted with indicated antibodies. Immunoreactive bands were revealed using ECL+ chemiluminescence kits (Amersham Biosciences) and analyzed with a ChemiDoc-XRS imaging station (Bio-Rad, Marnes-la-Coquette, France).

### Co-immunoprecipitation assay

Protein lysates prepared in ice-cold lysis buffer or conditioned media were subjected to immunoprecipitation assay using the Pierce Co-IP kit (Thermo Fisher Scientific) according to the manufacturer's protocol. 10 μg of antibody (anti CD47, anti-CD36, anti-FGF2, anti-VEGF, anti-β1 integrin and anti-LRP1 heavy chain) or non-specific IgGs were incubated with the delivered resin and covalently coupled. The antibody-coupled resin was incubated with whole HUVEC protein lysate overnight at 4°C under gentle agitation (no cross-linker was used to stabilize protein complexes before HUVEC lysis). Then the resin was washed and the protein complexes bound to the antibody were eluted. Immunoprecipitated protein complexes were solubilized under denaturing and reducing conditions, heated to 100°C for 5 min and analyzed by immunoblotting as described above.

### Wound healing assay

HUVECs were allowed to grow to confluence in 12-well plates and then incubated with 10 μg/mL mitomycin C at 37°C, 5% CO_2_ during 2 h to inhibit proliferation. The monolayer was wounded by scratching with a 200 μL pipette tip. Images were collected at different times using a phase contrast microscope with a 20× objective coupled to a Nikon digital camera. Wound area was quantified using ImageJ software (3 photographs/wound and 3 replicates for each condition).

### Transwell assay

24-well Transwell cell growing chambers (8.0 μm pore size; Greiner Bio-one, Dutscher) were used to assess endothelial cell migration. HUVECs were seeded into the upper compartment and incubated at 37°C for 12 h, the lower chamber containing complete EGM-2 with 2% (v/v) FBS acting as the stimulus for migration. Non-migrated cells on the top side of the surface were eliminated with a cotton swab, then the migrated cells at the bottom of the membrane were fixed with methanol, stained with Hoechst 33342 and visualized using a Zeiss inverted microscope (20× objective). Migration was quantified in 4 random microscopic fields per well using ImageJ software.

### Endothelial tube formation assay

Pre-chilled 24-well plates were coated with 250 μL matrigel (BD Biosciences) and incubated at 37°C for 30 min. HUVECs were plated at subconfluent densities in endothelial cell growth medium to form capillary-like structures. Pseudo-tubes formation was observed after 8 h incubation period using a Zeiss phase-contrast inverted microscope (20× objective; 4 photographs and 3 replicates for each condition). Total network length was quantified using ImageJ software. Branching points, nodal structures and tubes were assessed using the S.CORE web-based system for automatic image analysis (S.CO LifeScience, Hoehenkirchen, Germany).

### Small interfering RNA transfection

HUVECs were transfected with a pool of 3 target-specific 20–25 nt TSP-1 siRNAs (Santa Cruz Biotechnology, #SC-36665) designed to knock-down gene expression. As a control, cells were treated with an equal amount of nonspecific control RNA (Dharmacon, Thermo Fisher Scientific). Transfection of synthetic RNA (200 nM siRNA or control) was done using Amaxa™ HUVEC Nucleofector™ kit according to the manufacturer's protocol (Lonza). Following transfection with siRNA, cells were incubated for 24 h followed by the conditions indicated.

### Reverse Transcription-PCR

RNA was extracted with RNeasy kit (Qiagen, Courtaboeuf, France) according to the manufacturer's instructions. RNA (100 ng) from each sample was transcribed to cDNA using Verso cDNA kit (AB-1453/B) from ThermoFisher Scientific. Previously described PCR primers for TSP-1 [17] and β-actin [59] were synthesized by Eurogentec (Angers, France). Numbers of cycles were adjusted to ensure that amplifications were in a linear range. An aliquot of each PCR product was subjected to 1.2% (w/v) agarose gel electrophoresis and visualized by staining with ethidium bromide.

### Intracellular cGMP measurement

HUVECs (1 × 10^4^ per well) were grown over 24 h in 96-well plates containing EGM-2 full growth medium and then serum-starved in EBM-2 containing 0.1% (v/v) BSA over additional 24 h. Cells were then pre-treated during 15 min with 100 μM TAX2 in EBM-2 without additives plus 0.1% (v/v) BSA before treatment with NO donor in presence of 1 mM IBMX as a phosphodiesterases inhibitor. After 10 min, intracellular cGMP levels were determined according to the manufacturer's instructions using an enzyme immunoassay kit (Amersham Biosciences).

### Animals

8-week-old female C57BL/6 mice (average body weight, 18–20 g) were purchased from Janvier Labs (Saint-Berthevin, France). 8-week-old female BALB/C *nu/nu* mice were supplied by Charles River Laboratories (L'Arbresle, France). Animals were kept in a room with constant temperature and humidity, and food and water were given *ad libitum*. Mice were acclimatized to our laboratory conditions for 1 week before starting the experiments. The *in vivo* experiments were conducted in conformity with institutional ethical guidelines of the University of Reims Champagne-Ardenne and the CNRS (Centre National de la Recherche Scientifique), in compliance with the European Directive 2010/63/UE. The French Ministry of Higher Education and Research approved protocols under references 02203.01 and 02204.01.

### Mouse aortic ring assay for endothelial cell sprouting

Thoracic aortae were excised from C57BL/6 mice and peri-adventitial fibro-adipose tissues were removed. Aortae were then cut into 1 mm rings, washed and transferred to 96-well tissue culture plates coated with 100 μL per well of matrigel. Explants were then overlaid with additional 50 μL of matrigel. After polymerization, endothelial growth medium was added and then renewed every day. Digital images were taken on day 6 for quantitative analysis of vascular endothelial outgrowth using ImageJ software.

### *In vivo* models

For the subcutaneous melanoma tumor model, suspensions of B16F1 cells (2.5 × 10^5^ cells in 100 μL RPMI-1640 medium) were subcutaneously injected into the left flank of different randomized series of syngeneic C57BL/6 mice (*n* = 8–10 per group), as previously described [60]. Intraperitoneal administrations of peptide (10 mg/kg mouse weight) or controls (physiological serum 0.9% (w/v) NaCl or a scrambled peptide) were performed at days 3, 5 and 7. In human pancreatic carcinoma xenograft experiments, 3 × 10^6^ MIA PaCa-2 cells/mouse were implanted subcutaneously into the left flank of BALB/C athymic nude mice, then TAX2 intraperitoneal treatments (10 mg/kg mouse weight) were performed 3 times a week during 4 weeks starting at day 10 after tumor cells inoculation. Tumor volume was determined according to v = 0.5 A × B^2^, where A denotes the largest dimension of the tumor and B represents the smallest dimension [61]. At days indicated, mice were sacrificed and tumors were surgically extracted and fixed in 4% paraformaldehyde for further morphologic studies and for magnetic resonance imaging, or frozen for immunohistochemistry. Mice were also weighed every 2–3 days and daily checked for any modification in their behavior. Loss of 15% body weight was an indication for euthanasia as well as severe tumor necrosis, as it was sometimes associated with skin ulceration that would result in loss of body fluid and/or infection.

### Histopathological analysis of tumors

Histological analysis of formaldehyde-fixed and paraffin-embedded tumors were performed on hemalun, phloxin and saffron (HPS) stained 3 μm thick sections prepared using routine histological methods. To assess tumor-associated microvessel density (MVD), CD31 immunostaining was performed on 5 μm thick cryosections using biotin-labeled secondary antibody and streptavidin-HRP AEC detection system (Microm Microtech, Francheville, France), followed by hematein counter-coloration. Negative controls were done omitting the primary antibody. The necrotic part relative to total tumor surface for each tumor slice as well as MVD were quantified using ImageJ software.

### *Ex vivo* MR imaging of tumors

Tumors were studied by magnetic resonance imaging (MRI) for visualization of tumor necrosis when the subcutaneous allografts reached a diameter of about 0.8 cm (day 12). NMR *ex vivo* studies were performed on an 11.7 Tesla Bruker Avance DRX 500 system equipped with a micro-imaging probe (Bruker Biospin, Germany). High-resolution *T_1_*-weighted images were obtained with the following parameters: TR = 300 ms, TE = 2.2 ms, 256 × 256 × 256 matrix and a 0.9 × 0.9 × 1.5 cm FOV. Image treatment, segmentation, tri-dimensional reconstruction and quantification were performed using ImageJ and Amira 5.4.3 (Visualization Sciences Group, Burlington, MA, USA) software.

### μCT imaging of mice

Animals were anesthetized by isoflurane (Forene, Abbott France, Rungis, France) inhalation (3% for induction and 1–1.5% for maintenance) and then after placed in a carbon fibers bed. CT images were acquired on a dedicated small animal μCT scanner (Skyscan 1076, Bruker, Kontich, Belgium) while continuously rotating the camera by 180° with the following parameters: 50 kV, 0.5 mm Al filter, 200 μA source current, 35 μm isotropic resolution, 180 ms exposure time, 4 projection images per 0.7° rotation step and a retrospective synchronization. The projections were reconstructed using a filtered backprojection algorithm using Skyscan software (NRecon, Skyscan). For tumor angiography analysis, an alkaline earth-based nanoparticulate contrast agent (Viscover ExiTron nano 12000, Miltenyi Biotec, Paris, France) was injected in the mouse tail vein. Mice were imaged during the next 30 min following injection, a period during which no reduction in contrast was observed [35]. Analysis of reconstructed images and quantification of the vascular network were performed using Amira 5.4.3 software.

### Statistical analysis

GraphPad Prism 5.0 software (GraphPad, La Jolla, CA) was used for all statistical analyses. Each result is representative of at least 3 independent experiments. Data are expressed as the mean ± SE. Significance was assessed using 2-tailed Student's *t* test and analysis of variance (*ANOVA*) for *in vitro* and *ex vivo* studies; *Mann-Whitney U* test was used for *in vivo* experiments. For all tests, statistical significance was assumed when *p* < 0.05 (*).

## SUPPLEMENTARY FIGURES AND VIDEOS



## Abbreviations

3TSR: three thrombospondin-1 type 1 repeats
CBD: carboxy terminal cell binding domain
ECM: extracellular matrix
LRP-1: low-density lipoprotein receptor-related protein-1
MRI: magnetic resonance imaging
MVD: microvessel density
SIRP-α: signal regulatory protein alpha
TSP-1: thrombospondin-1
VEGFR2: vascular endothelial growth factor receptor 2
μCT: micro-computed tomography.
