# Supplementary material for: Identification of TAX2 peptide as a new unpredicted anti-cancer agent
Source: Oncotarget. 2015 May 22;6(20):17981–8000. doi: 10.18632/oncotarget.4025 (PMC4627230; doi:10.18632/oncotarget.4025)
Supplement: Supplementary file 1 [file oncotarget-06-17981-s001.pdf]

SUPPLEMENTARY FIGURES AND VIDEOS

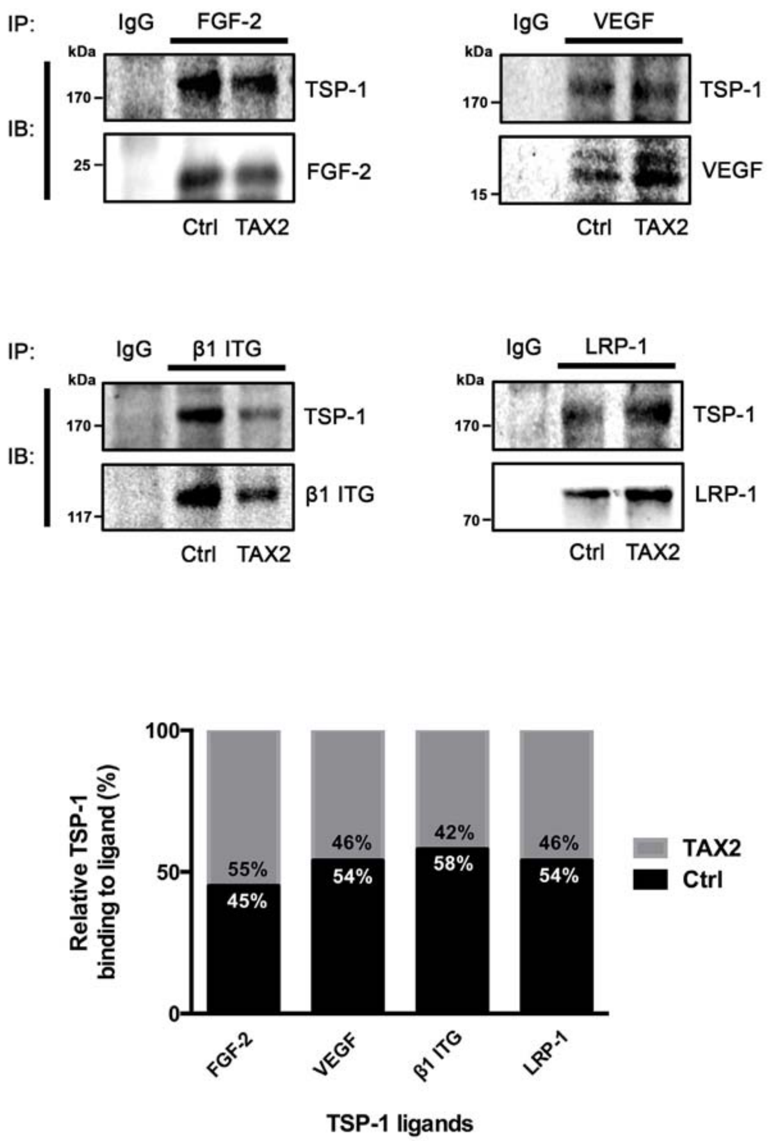

Supplementary Figure S1: TAX2 does not impede TSP-1 binding to soluble ligands or membrane bound receptors. HUVECs were treated with TAX2 (100 µM) or scrambled peptide (Ctrl, 100 µM). FGF-2 and VEGF were immunoprecipitated from conditioned media using anti-FGF-2 (clone C-18) and anti-VEGF (clone C-1), respectively. β1 integrin and LRP-1 were immunoprecipitated from total protein lysates using anti-β1 integrin (clone BV7) and anti-LRP-1 heavy chain (clone 8G1), respectively. In each case, non-specific IgGs were used as a negative control. Immunocomplexes were then submitted to SDS-PAGE and immunoblotted (IB) using specific TSP-1, FGF-2, VEGF, β1 integrin and LRP-1 light chain antibodies. Histogram represents the quantification of relative ligand-bound TSP-1 under TAX2 treatment as compared to control.

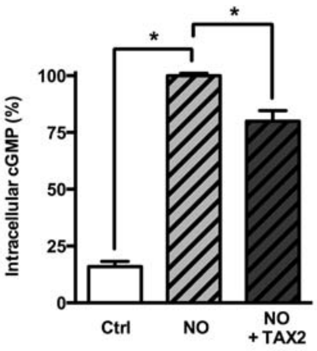

Supplementary Figure S2: TAX2 inhibits intracellular cGMP production by endothelial cells under NO stimulation. HUVECs (1×10^4 per well) were seeded in 96-well plates, maintained in EGM-2 growth medium for 24 h, and then serum-starved for an additional 24 h in EBM-2 containing 0.1% (v/v) BSA. Endothelial cells were then pre-treated with 100 µM TAX2 for 15 min before addition of 10 µM DEA-NONOate. After 10 min, intracellular cGMP was quantified as described in Material & Methods. Results are expressed as percent compared to control ($n = 3$, each performed in triplicate; *t* test; $*p < 0.05$).

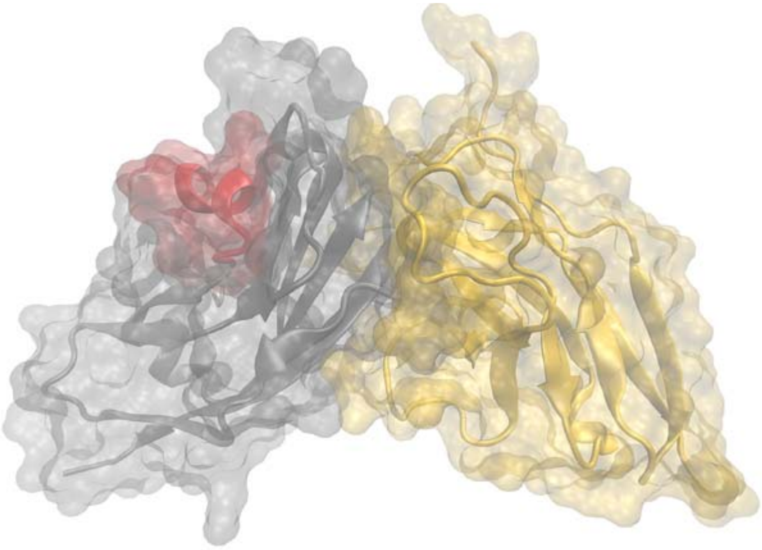

Supplementary Figure S3: CD47-derived TAX2 sequence is not located at the CD47:SIRPα molecular interface. Structure of CD47 ectodomain (*grey*, PDB ID code 2JJS, chain C) complexed with SIRPα (*yellow*, PDB ID code 2JJS, chain A), represented with a solvent-accessible surface on the proteins. Solvent-accessible surface of SQLLKGD sequence from which is derived TAX2 is highlighted (*red*).

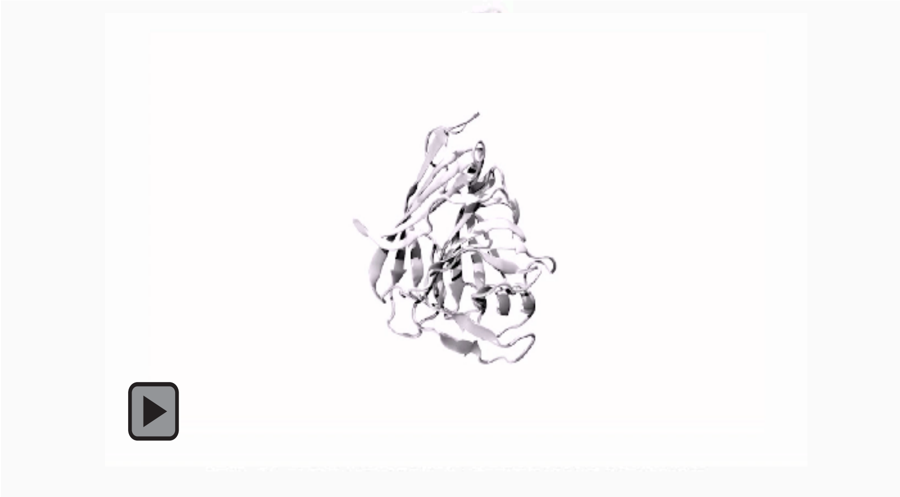

Supplementary Movie 1: TSP-1 CBD self-opening through R136 and D185 electrostatic scratch disruption. Normal mode analysis (MRMS = 2 Å along the mode 12) highlighted TSP-1 CBD large amplitude motions leading to the solvent exposure of the CD47-interacting sequence RFYVVMWK. Solvent accessible surface of TSP-1 CBD (*silver*) and RFYVVMWK interaction sequence (*red*) are highlighted, as well as lateral chains of the R136 and D185 residues allowing TSP-1 opening. Movie was generated from VMD files using FFmpeg software.

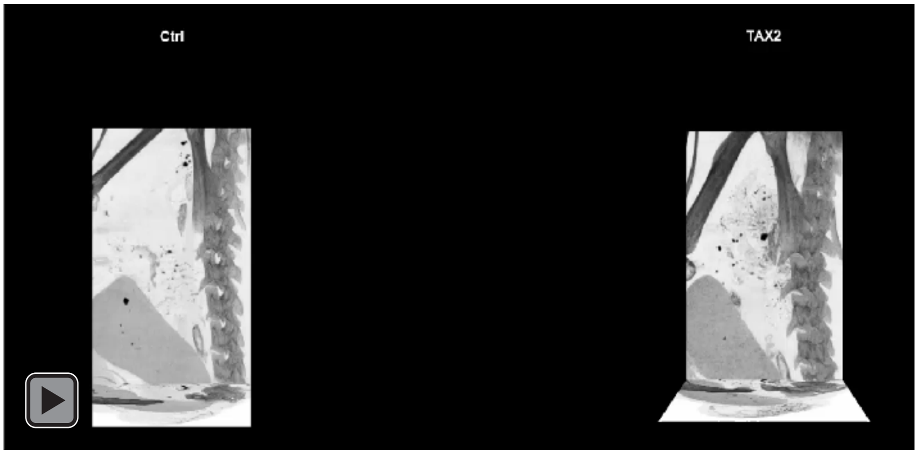

Supplementary Movie 2: Tumor angiography at day 14 after tumor challenge. As described for Figure 5A, mice were treated with scrambled (Ctrl, *left panel*) or TAX2 (*right panel*) peptide (10 mg/kg) before µCT imaging at day 14 after melanoma cells inoculation. Movies present the µCT data first as a volume rendering with a gray color map, then the region of the tumor is cropped out (*orange*) and processed with Amira software skeletonization tools. Thirdly, the extracted skeleton of the capillary network is visualized using a color-coded representation depending on structure thickness, in which thin structures are represented in blue, which changes to green and red when the vessel diameter increases. All data and movies were processed using Amira 5.4.3 software.
